# Therapeutic potential and mechanistic insights of astragaloside IV in the treatment of arrhythmia: a comprehensive review

**DOI:** 10.3389/fphar.2025.1528208

**Published:** 2025-04-10

**Authors:** Meilian Chen, Binlan Fu, Hao Zhou, Qiaomin Wu

**Affiliations:** ^1^ Cardiac and Pulmonary Department, Quanzhou Hospital of Traditional Chinese Medicine, Fujian, China; ^2^ Department of Internal Medicine, Chen Dai Central Health Center, Jinjiang, China; ^3^ Department of Cardiology, The 966th Hospital of The PLA Joint Logistic Support Force, Dandong, China; ^4^ Department of Cardiology, Guang’anmen Hospital, China Academy of Chinese Medical Sciences, Beijing, China

**Keywords:** arrhythmia, astragaloside IV, traditional Chinese medicine, *Astragalus membranaceus*, pharmacological mechanisms

## Abstract

Arrhythmia, a common cardiovascular disorder, results from disturbances in cardiac impulse generation and conduction, leading to decreased cardiac output and myocardial oxygenation, with potentially life-threatening consequences. Despite advancements in therapeutic approaches, the incidence and mortality associated with arrhythmia remain high, and drug-related adverse effects continue to pose significant challenges. Traditional Chinese Medicine (TCM) has attracted considerable attention for its potential as a complementary and alternative approach in treating cardiovascular diseases, including arrhythmia. Astragalus, a prominent herb in TCM, is commonly used in clinical practice for its multi-faceted therapeutic properties, encompassing anti-arrhythmic, cardiotonic, anti-inflammatory, and immunomodulatory effects. Astragaloside IV, a primary active compound in *Astragalus membranaceus*, has demonstrated cardioprotective effects through mechanisms such as antioxidant, anti-inflammatory, and anti-apoptotic activities. Although evidence suggests that astragaloside IV holds promise in arrhythmia treatment, comprehensive reviews of its specific mechanisms and clinical applications in arrhythmia are scarce. This review systematically explores the pharmacological properties and underlying mechanisms of astragaloside IV in arrhythmia treatment. Utilizing a targeted search of databases including PubMed, Web of Science, Cochrane Library, Embase, CNKI, and Wanfang Data, we summarize recent findings and examine astragaloside IV’s potential applications in arrhythmia prevention and treatment. Our analysis aims to provide a theoretical foundation for the development of novel arrhythmia treatment strategies, while offering insights into future research directions for clinical application.

## 1 Introduction

Arrhythmia arises from disturbances in pulse generation, conduction, or a combination of both, potentially leading to reduced cardiac output or myocardial oxygenation, conditions that can be life-threatening. It is commonly classified as tachycardia (supraventricular or ventricular) or bradycardia, with clinical manifestations varying according to the type, cardiac function, and degree of hemodynamic impact. While mild arrhythmias may exhibit no apparent symptoms, severe forms can induce palpitations, chest discomfort, dizziness, hypotension, and sweating. In extreme cases, syncope, Adams-Stokes syndrome, or even sudden death may occur. Notably, arrhythmia is frequently associated with other disorders in clinical settings, including coronary atherosclerotic heart disease, heart failure (Sossalla and Vollmann, 2018), stroke (Ruthirago et al., 2016), immune disorders (Gawałko et al., 2020), and psychological conditions like anxiety and depression. Thus, arrhythmia is not only a widespread issue within cardiovascular diseases but also an aspect that is often overlooked. Empirical research indicates that conditions such as heart failure, diabetic cardiomyopathy, age-related cardiac insufficiency, and genetic diseases are linked to an increased risk of malignant arrhythmia (Hamilton et al., 2021). One study noted a marked rise in arrhythmia-related hospitalizations, primarily due to atrial fibrillation (AF), from 1993 to 2013, exceeding the hospitalization rates for both heart failure and myocardial infarction (MI) (Gallagher et al., 2019). This evidence underscores the critical importance of addressing arrhythmia within the medical field.

The mechanisms of arrhythmia typically involve three primary pathways: enhanced automaticity, triggered activity, and reentry. These mechanisms are closely linked to pathological changes, including ion channel dysregulation, aging, hypoxic-stress injury, fibrosis, and autonomic nervous dysfunction (Schömig et al., 1995; Hool, 2005; García-Mendívil et al., 2022; Pironet et al., 2024). Current therapeutic strategies for arrhythmia include pharmacological treatments, surgical interventions, and lifestyle modifications. However, the incidence and mortality rates associated with arrhythmia continue to rise, and adverse drug effects remain a significant obstacle in clinical practice. Recently, traditional Chinese medicine (TCM) has garnered extensive attention for its potential in treating cardiovascular diseases, offering a range of benefits such as anti-arrhythmic, cardiotonic, diuretic, vasodilatory, and anti-atherosclerotic effects. Evidence increasingly suggests that TCM can serve as a valuable complementary and alternative approach for both the primary and secondary prevention of cardiovascular diseases (Hao et al., 2017).

Astragalus, a widely used traditional Chinese medicinal herb, is incorporated into various herbal formulations for its numerous therapeutic effects, including immune modulation, anti-inflammatory (D’Avino et al., 2023), antiviral (Ghabeshi et al., 2023), and anticancer properties (Hwang et al., 2021). Astragalus is frequently used to address various cardiovascular conditions, including arrhythmia, coronary atherosclerotic heart disease, and hypertension. The plant contains over 100 distinct compounds, such as flavonoids, saponins, polysaccharides, and amino acids (Guo et al., 2019). Astragaloside IV (AS-IV), a key active constituent in the aqueous extract of *Astragalus membranaceus*, is classified as a pentacyclic triterpenoid and exhibits several pharmacological activities, including antioxidant, anti-inflammatory, and anti-apoptotic effects. Studies have shown that AS-IV has beneficial effects in areas such as neuroprotection, hepatoprotection, anticancer, and antidiabetic applications (Zhang et al., 2020) and also provides protective effects against arrhythmia. However, research on the specific mechanisms of AS-IV in arrhythmia remains limited, and a comprehensive review is lacking.

In this article, we conduct a systematic review of existing studies on the pharmacological effects and mechanisms of AS-IV in the treatment of arrhythmia. Using the keywords “astragaloside,” “*cardi*,” “*arrhy*,” “fibrillation” and “flutter,” we searched databases including PubMed, Web of Science, Cochrane Library, Embase, China National Knowledge Infrastructure, and Wanfang Data. We aim to summarize the current understanding of AS-IV’s therapeutic potential and innovative applications in the prevention and treatment of arrhythmia. Through this review, we aspire to provide a theoretical foundation for the development of novel treatment strategies for arrhythmia and to offer guiding insights for future clinical research.

## 2 Pharmacokinetic and pharmacodynamic properties

### 2.1 Molecular structure and physicochemical properties of AS-IV

AS-IV is classified as a pentacyclic triterpenoid compound. It is a cyclic derivative of astragaloside, with β-D-xylopyranosyl and β-D-glucopyranosyl residues attached at the O-3 and O-6 positions, respectively (Xu et al., 2023). AS-IV is a white powder with the molecular formula C_41_H_68_O_14_ and a relative molecular weight of 784.97. Its CAS registry number is 84687–43-4, and it has a melting point between 299°C and 300°C when dissolved in methanol. Structurally, AS-IV shares similarities with steroid drugs, characterized by an extremely low aqueous solubility. It demonstrates high solubility in polar organic solvents such as methanol, ethanol, and acetone, while its solubility is significantly reduced in weakly polar organic solvents like chloroform or ethyl acetate (Tan et al., 2020).

### 2.2 Characteristics of pharmacokinetics

AS-IV demonstrates pronounced linear pharmacokinetic properties in preclinical studies. Research has shown that the area under the concentration-time curve (AUC) correlates linearly with the administered dose, and no significant differences in the pharmacokinetics of AS-IV have been observed between rats and Beagle dogs (Zhang et al., 2007). Clinical studies further support these findings, reporting mean maximum plasma concentrations (Cmax) of AS-IV at 2.12, 3.59, 3.71, and 5.17 μg/mL following single doses of 200, 300, 400, and 500 mL of astragaloside injection (AI), respectively (Xu et al., 2013). Corresponding AUC values (AUC(0-∞)) were measured at 4.38, 9.75, 13.59, and 18.22 μg h/mL, while elimination half-lives (t1/2) were recorded as 2.14, 2.59, 2.62, and 2.69 h. In repeated-dose studies, Xu et al. (2013) reported no significant variations in key pharmacokinetic parameters—peak time (Tmax), t1/2, and AUC—between Day 1 and Day 7, suggesting stable pharmacokinetics with prolonged administration. Following a 500 mL dose of AI, 3.91% of the administered AS-IV was excreted in the urine within a 24-h period (Xu et al., 2013). Co-administration with other compounds has been shown to enhance AS-IV pharmacokinetics. For example, interaction with atractylenolide I led to increased oral Cmax and systemic plasma exposure of AS-IV (Song et al., 2014).

### 2.3 Research on medication dosage

The content of AS-IV in Radix Astragali varies significantly depending on its source (Liu et al., 2009), and sample preparation methods also impact its concentration (Monschein et al., 2013). *In vivo* experiments have categorized 5 mg/kg/day as a moderate dose, while 10 mg/kg/day and 2.5 mg/kg/day are considered high and low doses, respectively (Zhang and Chen, 2013). Effective dosages reported across studies differ; for example, administration of AS-IV at 0.3 and 1.0 mg/kg/day improved cardiac function in a rat model of heart failure (Zhao et al., 2009). Additionally, Qiu et al. (2010) demonstrated that AS-IV at 50 and 100 mg/mL significantly improved the function of homocysteine-induced human umbilical vein endothelial cells by reducing reactive oxygen species (ROS) accumulation and increasing superoxide dismutase (SOD) activity (Qiu et al., 2010). (see Table 1)

**TABLE 1:** Research on dosage of AS-IV

Dose	Experimental type	Experimental model	Mechanism	Reference
20 mg/L	*in vitro*	hADSCs in HG	Pink1/Parkin↑	Jun-li et al. (2023), Sun et al. (2023)
10, 50, 100 μmol/L	*In vitro*	BLM-induced VSMC senescence model	Parkin↑	Li et al. (2022a)
20, 40, 80 mg/kg	*In vivo*	D-gal-induced vascular aging model, BABL/C	Parkin↑	Li et al. (2022a)
50 μmol/L	*in vitro*	PC12	GSK-3β/mPTP↓	Fu et al. (2020)
5 mg/kg	*In vivo*	bradycardia Model, SD	Klotho/HCN4↑	Qiu et al. (2016)
40 μmol/L	*in vitro*	mouse podocytes	HO-1/GCLC/GCLM↑	Shen et al. (2023)
20, 40, 80 mg/kg 0, 20, 40, 80, 120 μmol/L	*in vitro*/*In vivo*	diabetes rat model PA-treated H9c2	CD36↓	Li et al. (2023)
10 μmol/L 50, 100 mg/kg	*in vitro*/*In vivo*	3T3-L1, HFD-treated ICR	cAMP↓, p-Akt↑	Du et al. (2018)
12.5 mg/kg, 25 mg/kg, 50 mg/kg	*In vivo*	HFD-treated C57BL/6	FXR↓, GLP-1↑	Zhai et al. (2022)
50 μmol/L	*in vitro*	H9c2	NO/cGMP/PKG↑,GSK-3β↓, mPTP↓	He et al. (2012)
40 μmol/L	*in vitro*	SD-CMs	Hes1↑	Huang et al. (2016)
40 mg/kg	*In vivo*	SD Rat MI/R Model	Keap1/Nrf2/HO-1↑	Jiang et al. (2019)
5 mg/kg 10, 20, 40, 80 μmol/L	*in vitro*/*In vivo*	SD Rat heart A/R Model, H9c2	Bcl-2/MMP↑, ROS/mPTP/cyt c↓	Luo et al. (2019)
0.1/1 μmol/L	*in vitro*	GP Cardiomyocytes	*I*_CaL_↑	Zhao et al. (2015a)
30 μmol/L	*in vitro*	NRCMs	PKA-Cα↑, mRNASer16-PLN↑	Zhang et al. (2012)
0.1, 1, 10 μmol/L	*in vitro*	GP Cardiomyocytes	IK↓, *I*_CaL_↓	Zhao et al. (2013)
300 mg/L	*in vitro*	CVB3-DCM	TGF-β1↓	Chen et al. (2011)
10, 50 ng/mL	*in vitro*	H9c2	P38↓, JNK↓,ERK↓	Sun et al. (2021)

Abbreviation: hADSCs, human adipose-derived stem cells; HG, high glucose; BLM, bleomycin; VSMC, vascular smooth muscle cell; D-gal, D-galactose; PA, palmitic acid; HFD, high-fat diet; CMs, cardiomyocytes; MI/R, myocardial ischemia/reperfusion; A/R, anoxia/reoxygenation; GP, guinea pig; NRCMs, neonatal rat cardiomyocytes; DCM, dilated cardiomyopathy.

Meta-analyses have shown that AS-IV provides significant cardioprotective effects by reducing cardiac preload and afterload and inhibiting cardiac hypertrophy, with an effective therapeutic dose range of 10–80 mg/kg/day (Zhang et al., 2023). A potential nonlinear positive correlation between dosage and therapeutic efficacy has also been suggested (Zhang et al., 2023). Furthermore, AS-IV’s cardioprotective effects in heart failure appear to be dose-dependent, as demonstrated by increased expression of nuclear factor erythroid 2-related factor 2 (Nrf2)/antioxidant response element (ARE)-dependent genes, such as heme oxygenase-1 (HO-1), NAD(P)H: quinone oxidoreductase 1 (NQO-1), and sulfiredoxin-1 (Srxn-1), with rising AS-IV concentrations in the oxygen-glucose deprivation model (Gu et al., 2015). Animal studies confirm that AS-IV exhibits no evident toxicity or adverse effects within a safe dose range, equivalent to 35–70 times the human dose (Yu et al., 2007).

A pharmacokinetic study in 2013 investigated AS-IV’s tolerability among healthy Chinese participants following single and multiple intravenous infusions of AI. The Cmax of AS-IV after administration of 200, 300, 400, and 500 mL was 2.12, 3.59, 3.71, and 5.17 μg/mL, respectively (Xu et al., 2013). The AUC(0-∞) values were 4.38, 9.75, 13.59, and 18.22 μg h/mL, and the elimination t1/2 averaged 2.14, 2.59, 2.62, and 2.69 h, respectively (Xu et al., 2013). Within 24 h of administering 500 mL of AI, cumulative urinary excretion of AS-IV reached 3.91%, indicating linear pharmacokinetics within a 200–500 mL dose range, with no accumulation observed following once-daily administration of AI (Xu et al., 2013). Additionally, another study reported that following intravenous injection, the primary distribution of Astragaloside IV occurred in the lungs, kidneys, heart, stomach, and spleen, which may reflect the selective uptake of the compound by these organs (Ya et al., 2019).

Caution is advised in pregnant women, as fetotoxic effects have been documented in rats and rabbits at doses exceeding 0.5 mg/kg, though no teratogenic effects have been observed (Jiangbo et al., 2009).

### 2.4 Degree of oral bioavailability

The oral bioavailability of AS-IV is notably low, with studies showing it to be 2.2% in rats (Gu et al., 2004) and 7.4% in Beagle dogs (Zhang et al., 2007). This limited bioavailability is primarily attributed to AS-IV’s relatively large molecular weight, low lipophilicity, and poor intestinal permeability, resulting in suboptimal efficacy for single oral administrations (Artursson and Karlsson, 1991). However, strategies to enhance the oral bioavailability of AS-IV have shown promise. For instance, the AS-IV inclusion complex demonstrated increased bioavailability in rats, with values reaching 10.2%–11.2%, compared to 3.2% for an aqueous solution (Jun-Xian et al., 2011). Complexation with 2-hydroxypropyl-β-cyclodextrin also significantly enhanced AS-IV bioavailability in rats (Chen and Gu, 2009).

Additionally, derivatives of AS-IV, such as LS-102, have shown enhanced pharmacological activity at comparable concentrations (Li Z. et al., 2022). Another derivative, HHQ16, exhibited substantial efficacy in improving cardiac function, notably increasing the left ventricular ejection fraction (LVEF) and left ventricular shortening fraction (LVFS) at a dose of 10 mg/kg. Its effects were observed to surpass those of commonly used drugs, including enalapril and LCZ696 (Wan et al., 2023). However, it is important to note that these findings are largely limited to animal studies, and clinical evidence remains scarce. These observations highlight the challenges associated with AS-IV’s oral administration and underscore the potential of formulation strategies in enhancing its therapeutic efficacy.

## 3 Mechanism of AS-IV in arrhythmia

**FIGURE 1:**
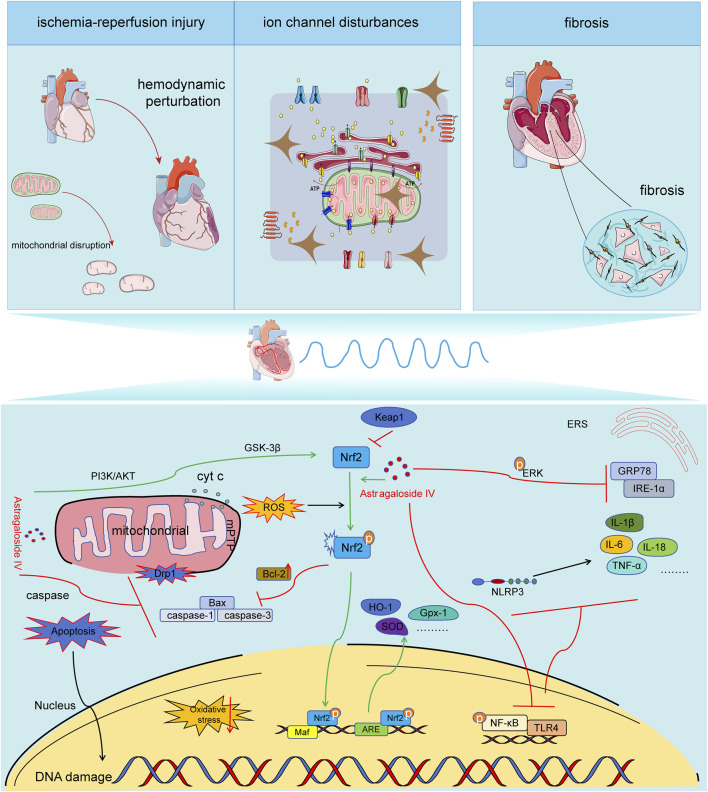
Mechanisms of AS-IV in the pathogenesis of arrhythmia. Astragaloside IV exhibits anti-ischemia-reperfusion injury, anti-ion channel disorder, and anti-myocardial fibrosis effects through maintaining mitochondrial quality control, counteracting oxidative stress, exerting anti-inflammatory actions, and resisting apoptosis. It is capable of inhibiting the expression of Drp1, decreasing the release of Cyt C, influencing the opening of mPTP pores, suppressing mitochondrial fission and fragmentation, and preserving the integrity of mitochondrial structure and function. It can activate the PI3K/AKT//Nrf2/ARE signaling pathway, enhance the expression and nuclear translocation efficiency of Nrf2, initiate the expression of downstream antioxidant factors, and alleviate oxidative stress. It can restrain the activation of the NF-κB/TLR4/NLPR3 signaling pathway, reduce the expression of downstream inflammatory factors, and mitigate inflammation. It can inhibit the caspase pathway, regulate the expression of Bcl-2/bax, and decrease cardiomyocyte apoptosis.

The underlying pathological mechanisms of arrhythmia include ischemia-reperfusion injury, fibrosis, and ion channel dysfunction, all of which disrupt the normal electrical conduction and automaticity of the heart. These conditions contribute to the initiation and progression of arrhythmic events, which can lead to serious clinical outcomes. Emerging evidence suggests that AS-IV exerts therapeutic effects by ameliorating I/R injury, reducing fibrosis, and restoring ion channel homeostasis, thus offering a promising approach for arrhythmia management (Table 2). This section delves into the mechanistic pathways through which AS-IV modulates these critical pathological processes (Figure 1).

**TABLE 2:** Therapeutic effect and mechanism of AS-IV on arrhythmia.

Treatment effect	Mechanism	Reference
Against I/R Injury	Collagen I↓, Collagen III↓, HYP↓	Shi et al. (2021)
	IGF1R/ATP↑	He et al. (2022)
	PI3K/AKT/GSK-3β↑	Wei et al. (2019)
	GATA-4/Bcl-2/Beclin-1↑	Yang et al. (2020)
	p-Akt/Akt↑, Bcl-2/bax/caspase-3↓	Jia-Lin et al. (2017) Lu et al. (2015a) Chen et al. (2020)
	TLR4//NF-κB↓	Lu et al. (2015a)
	Keap1/Nrf2/HO-1↑	Jiang et al. (2019)
	PI3K/Akt/GSK↑	Chen et al. (2020)
Against collagen and fibrosis	ROS/NLRP3/Caspase-1↓	Zhang et al. (2022)
	Nrf-2/HO-1↑, IL-1β↓,IL-18↓	Chen et al. (2023)
	Smad4/TGF-β1/ECM↓	Chen et al. (2011)
	TRPM7/TGF-β/Smads↓	Wei et al. (2020)
	Akkermansia↑,Defluviitaleaceae_UCG-011↑, Rikenella↑	Du et al. (2022)
Antagonism targeting ion channel disorders	*I*_*f*_↑,HCN4↑	Liu et al. (2018)
	ROS/calpain-1/Ca^*2+*^↓	Mei et al. (2015)
	cAMP/PKA/PLN/SERCA2a↑	Zhang et al. (2012)
	*I*_CaL_↓,*I*_*K*_↓,APD↑	Zhao et al. (2013)
	CaSR/Ca^*2+*^/CaMKII/CaN↓	Lu et al. (2018)
	p-ERK1/2↑	Yin et al. (2019)
	mitoKATP↑	Guan et al. (2015)

Abbreviation: HYP, hydroxyproline; IGF1R, insulin-like growth factor 1 receptor; GATA-4, GATA-binding protein 4; TLR4, toll-like receptor 4; Keap1, kelch-like ECH-associated protein 1; Nrf2, nuclear factor erythroid 2-related factor 2; HO-1, heme oxygenase-1; GSK, glycogen synthase kinase; NLRP3, NOD-like receptor family pyrin domain containing 3; TGF-β1, transforming growth factor-β1; TRPM7, transient receptor potential cation channel, subfamily M, member 7; ECM, excessive deposition of extracellular matrix; HCN, hyperpolarization-activated cyclic nucleotide-gated channels; cAMP, cyclic adenosine monophosphate; PKA, protein kinase A; PLN, phospholamban; SERCA2a, sarcoplasmic/endoplasmic reticulum calcium ATPase 2a; APD, action potential duration; CaSR, calcium sensing receptor; CaMKII, Ca2+/calmodulin-dependent protein kinase II; CaN, calcineurin; mitoKATP, mitochondrial ATP-sensitive potassium channel.

### 3.1 The resistance mechanism against ischemia-reperfusion injury

I/R injury remains a significant challenge in myocardial reperfusion therapy. The resultant myocardial cell damage and electrophysiological alterations are strongly predisposed to triggering arrhythmias, particularly ventricular arrhythmias such as premature ventricular beats, ventricular tachycardia, and ventricular fibrillation. These conditions can lead to severe hemodynamic disturbances and, in extreme cases, sudden cardiac death (Dauerman and Ibanez, 2021). A growing body of evidence suggests that AS-IV exerts protective effects against cardiac I/R injury through various mechanisms, including enhancing circulation, preventing cellular apoptosis, mitigating oxidative stress, and exerting anti-inflammatory actions (Zheng et al., 2018). A comprehensive meta-analysis demonstrates that AS-IV significantly improves LVEF, reduces left ventricular end-diastolic pressure, downregulates myocardial creatine kinase and troponin expression, optimizes ST segment depression, and minimizes the MI area (Zheng et al., 2018). Furthermore, Shi et al. (2021) revealed that AS-IV alleviates hemodynamic abnormalities in rats with acute MI by suppressing the deposition of type I and type III collagen and regulating myocardial hydroxyproline levels (Shi et al., 2021).

He et al. (2022) have shown that AS-IV enhances ATP production through activation of the insulin-like growth factor 1 receptor signaling pathway. This process reduces endothelial junction protein expression, thereby mitigating albumin leakage from endothelial cells and reducing white blood cell infiltration after I/R, which alleviates cellular edema and enhances coronary vascular endothelial barrier integrity, as well as minimizing microvascular leakage (He et al., 2022). In a rat myocardial I/R model, AS-IV administration significantly improved left ventricular systolic pressure, FS, and EF, while decreasing left ventricular end-diastolic pressure. Moreover, serum lactate dehydrogenase and creatine kinase levels were significantly reduced, and the heart-to-body weight ratio and MI area were also decreased. The primary mechanism underlying these effects involves AS-IV’s ability to increase phosphorylation of PI3K/AKT and GSK-3β proteins, thereby activating the PI3K/AKT/GSK-3β signaling pathway to protect cardiomyocytes from apoptotic damage induced by I/R (Wei et al., 2019).

In an *in vitro* H9c2 cell model subjected to hypoxia/reoxygenation, AS-IV upregulated the expression of GATA-binding protein 4 (GATA-4), Bcl-2, and P62, while downregulating apoptosis and autophagy-associated genes such as PARP, caspase-3, and Beclin-1. These findings suggest that AS-IV inhibits apoptosis and autophagy in H9c2 cells exposed to I/R. Notably, the expression of GATA-4 was shown to enhance the interaction between Bcl-2 and Beclin-1 under I/R stress, which may contribute to cell survival (Yang et al., 2020). Additionally, another study demonstrated that AS-IV upregulates p-Akt/Akt and Bcl-2 expression in a concentration-dependent manner while downregulating pro-apoptotic proteins such as Bax and caspase-3, thereby mitigating I/R-induced apoptosis (Jia-lin et al., 2017).

I/R injury induces intense local and systemic inflammatory responses, which exacerbate tissue damage and impede left ventricular recovery (Algoet et al., 2023). Studies indicate that AS-IV significantly suppresses the TLR4/NF-κB signaling pathway and downregulates the expression of inflammatory cytokines such as TNF-α and IL-1β. Furthermore, AS-IV administration reduces the expression of Caspase-3 and Bax while enhancing Bcl-2 levels. These effects correlate with a reduction in infarction size and alterations in myocardial morphology, suggesting that AS-IV’s anti-apoptotic effects in the early stages of reperfusion contribute to mitigating subsequent myocardial necrosis. These findings imply that AS-IV counteracts inflammation-mediated cell apoptosis induced by I/R (Lu M. et al., 2015).

Oxidative stress plays a pivotal role in the pathogenesis of myocardial I/R injury (Peoples et al., 2019). During myocardial reperfusion following ischemia, the production of oxygen free radicals increases dramatically, disrupting oxidative balance and antioxidant defense systems, leading to cellular damage and abnormal immune responses (Eltzschig and Eckle, 2011). Jiang et al. (2019) demonstrated that AS-IV activates the Keap1/Nrf2 antioxidant pathway by downregulating Keap1 expression in the cytoplasm, promoting dissociation of Keap1 from Nrf2, facilitating Nrf2 release and translocation to the nucleus, where it binds Maf to initiate the transcription of the antioxidant gene HO-1. This activation scavenges ROS, reduces the consumption of metabolic substrates like succinate, and mitigates excessive ROS production and lipid peroxidation, thereby exerting an antioxidant effect on the myocardium (Jiang et al., 2019).

Mitochondrial dysfunction is a hallmark of myocardial I/R injury, with impaired mitochondrial quality control serving as a critical factor in arrhythmia development (Bai et al., 2023). Studies have shown that after I/R injury, a reduction in ATP 5D protein and its mRNA occurs, leading to decreased ATP/ADP and ATP/AMP ratios, elevated levels of P-MLC2 and serum cTnI, and subsequent cardiac dysfunction. These changes can be significantly alleviated by pretreatment with AS-IV (Tu et al., 2013). Chen et al. (2021) demonstrated that AS-IV derivative LS-102 protects left ventricular function in I/R rats, reducing arrhythmia incidence and infarct size. The mechanism involves activation of the PI3K/Akt/GSK-3β signaling pathway, which reduces Drp1 phosphorylation at Ser616 while enhancing its phosphorylation at Ser637, thereby inhibiting Drp1-mediated mitochondrial fission. This prevents mitochondrial fragmentation, promotes mitochondrial elongation, and preserves mitochondrial structural integrity and function. Additionally, LS-102 stabilizes mitochondrial membrane potential, reduces the Bax/Bcl-2 ratio, and suppresses Caspase-3 expression, thereby alleviating mitochondrial fission-mediated apoptosis (Chen et al., 2020).

In conclusion, AS-IV mitigates cardiac I/R injury through multiple molecular mechanisms, including anti-apoptotic, anti-inflammatory, antioxidant, and mitochondrial protective effects. These actions collectively reduce the incidence of arrhythmias and improve myocardial recovery post-I/R.

### 3.2 Resistance against collagen and fibrosis

Myocardial fibrosis is a prominent pathological alteration observed in numerous cardiovascular diseases, serving as a critical pathophysiological mechanism in cardiac remodeling. Upon injury to cardiomyocytes, fibroblasts proliferate extensively and differentiate into myofibroblasts, which subsequently secrete significant amounts of collagen. This excessive deposition of extracellular matrix (ECM) proteins leads to abnormal accumulation, reducing myocardial compliance, increasing cardiac stiffness, and ultimately impairing cardiac function (Kong et al., 2014). Atrial fibrosis is recognized as a structural remodeling marker associated with arrhythmogenesis (Scholz et al., 2019). Studies indicate that excessive collagen deposition promotes atrial tissue fibrosis, which disrupts the propagation of myocardial impulses and raises the risk of arrhythmia (Akhurst and Hata, 2012).

Research has demonstrated that AS-IV significantly alleviates cardiac hypertrophy and fibrosis induced by MI in mice. The underlying mechanism involves AS-IV’s ability to reduce mitochondrial ROS in myocardial cells of MI mice, inhibit NLRP3 inflammasome activation, and subsequently downregulate the expression of downstream inflammatory factors such as IL-18 and IL-1β, along with the caspase-1 regulator involved in cell pyroptosis. This modulation alters the expression of the key structural region of GSDMD-N in cell pyroptosis, thereby effectively improving MI-induced cardiac hypertrophy and fibrosis (Zhang et al., 2022). Furthermore, Chen et al. found that AS-IV can activate the Nrf-2/HO-1 signaling pathway, suppressing the expression of downstream inflammatory factors like IL-1β and IL-18, mitigating the DOX-induced inflammatory response, and reducing the extent of myocardial fibrosis (Chen et al., 2023).

TGF-β1 is widely acknowledged as a potent inducer of collagen production by cardiac fibroblasts (Euler-Taimor and Heger, 2006), playing a crucial role in myocardial hypertrophy and fibrosis by activating fibroblasts and promoting collagen synthesis (Annes et al., 2003). Chen reported that AS-IV can inhibit the expression of Smads2/3, particularly Smad4, thus blocking TGF-β1 downstream signaling and down-regulating procollagen gene expression. This results in decreased synthesis of ECM components, significantly reducing abnormal collagen deposition in myocardial tissue and substantially reversing myocardial hypertrophy and fibrosis (Chen et al., 2011). Further research by Yang et al. revealed that AS-IV inhibits the TRPM7/TGF-β/Smads signaling pathway, reduces TRPM7 current, lowers calcium ion activity, and thereby attenuates the progression of myocardial fibrosis (Wei et al., 2020). Intriguingly, Du et al. found that gut microbiota alterations are closely related to cardiac fibrosis. Their study showed that AS-IV enhances the abundance of gut microbiota such as *Akkermansia*, *Defluviitaleaceae_UCG-011*, and *Rikenella* in ISO-induced heart failure model mice. This modulation decreases the metabolism of the cardiovascular risk factor phenylalanine, ultimately reducing myocardial collagen deposition, lowering the cardiac weight index, slowing the progression of myocardial fiber thickening and necrosis, and preventing and ameliorating ISO-induced myocardial fibrosis (Du et al., 2022).

### 3.3 Antagonism targeting ion channel disorders

Oxidative stress and inflammatory processes accelerate the development of atrial fibrosis, leading to increased collagen deposition and structural remodeling of the atria. These alterations impact the functionality of various ion channels, including hyperpolarization-activated cyclic nucleotide-gated (HCN) channels, L-type calcium channels, sodium channels, and potassium channels. Such channel modifications alter ion flow, transmembrane potential, and reduce both APD and conduction velocity, ultimately disrupting cardiac electrophysiological activity and elevating the risk of arrhythmias (Karam et al., 2017).

The HCN channel, also known as the If channel, is activated near the end of the repolarization phase, enabling inward currents mediated by Na^+^ and K^+^ throughout diastolic depolarization (Rivolta et al., 2020). HCN4 is a primary component of the sinoatrial node current, accounting for 70%–80% of the If current, and is implicated in various cardiovascular conditions, including sinoatrial node dysfunction and arrhythmias (Baruscotti et al., 2010; Schweizer et al., 2014). Liu et al. reported that AS-IV can enhance the If current density in I/R-injured sinoatrial node cells, shorten APD at 20% and 50% repolarization in SAN cells, and upregulate HCN4 expression, thus helping maintain cell volume and cytoskeletal integrity in SAN cells, with potential therapeutic effects for sick sinus syndrome (Liu et al., 2018).

Calcium ion imbalance is a key contributor to the onset and progression of arrhythmia (Deo et al., 2017). Meng et al. demonstrated that AS-IV reduces ROS accumulation in ISO-induced cardiomyocytes, mitigates mitochondrial membrane damage, counters Ca^2+^ redistribution due to oxidative stress, inhibits calpain-1 activation, maintains calcium homeostasis, and prevents apoptosis in ISO-induced myocardial hypertrophy, reducing arrhythmia risk (Mei et al., 2015). Zhang et al. showed that AS-IV activates the cAMP/PKA pathway, increasing phospholamban (PLN) phosphorylation at Ser16 and relieving PLN-mediated inhibition of sarcoplasmic reticulum Ca^2+^ ATPase 2a, thereby reducing spontaneous Ca^2+^ release in post-I/R mice and lowering the risk of AF (Zhang et al., 2012). Zhao et al. reported that AS-IV reduces the amplitude of L-type Ca^2+^ current in guinea pig ventricular cells in a concentration-dependent manner, influencing L-type Ca^2+^ channel kinetics, suppressing channel activity, and blocking Ca^2+^ current, while concurrently inhibiting K^+^ current to prolong APD (Zhao et al., 2013). Lu et al. revealed that AS-IV inhibits the Ca2^+^/CaMKII/CaN pathway by attenuating calcium-sensing receptor sensitivity, reducing Ca^2+^ release from the sarcoplasmic reticulum, and decreasing intracellular Ca^2+^ to prevent calcium overload (Lu et al., 2018). Similarly, Yin et al. hypothesized that AS-IV reduces calcium overload by inhibiting galanin receptor activity, enhances ERK1/2 phosphorylation, and mitigates cardiomyocyte apoptosis induced by I/R (Yin et al., 2019). These findings suggest AS-IV’s critical role in regulating calcium channel activity and maintaining intra- and extracellular calcium balance.

Beyond calcium homeostasis, AS-IV also modulates dysfunctions in other ion channels. For instance, AS-IV prolongs action potential duration in guinea pig ventricular myocytes by inhibiting delayed rectifier K^+^ channels and enhancing inward rectifier K^+^ channels (Zhao et al., 2013). Studies further indicate that in advanced I/R stages, AS-IV activates mitochondrial ATP-sensitive K^+^ channels, leading to depolarization, reduced mitochondrial Ca^2+^ uptake, and decreased mitochondrial calcium overload (Guan et al., 2015).

## 4 Synergistic effects and mechanisms of AS-IV

**FIGURE 2:**
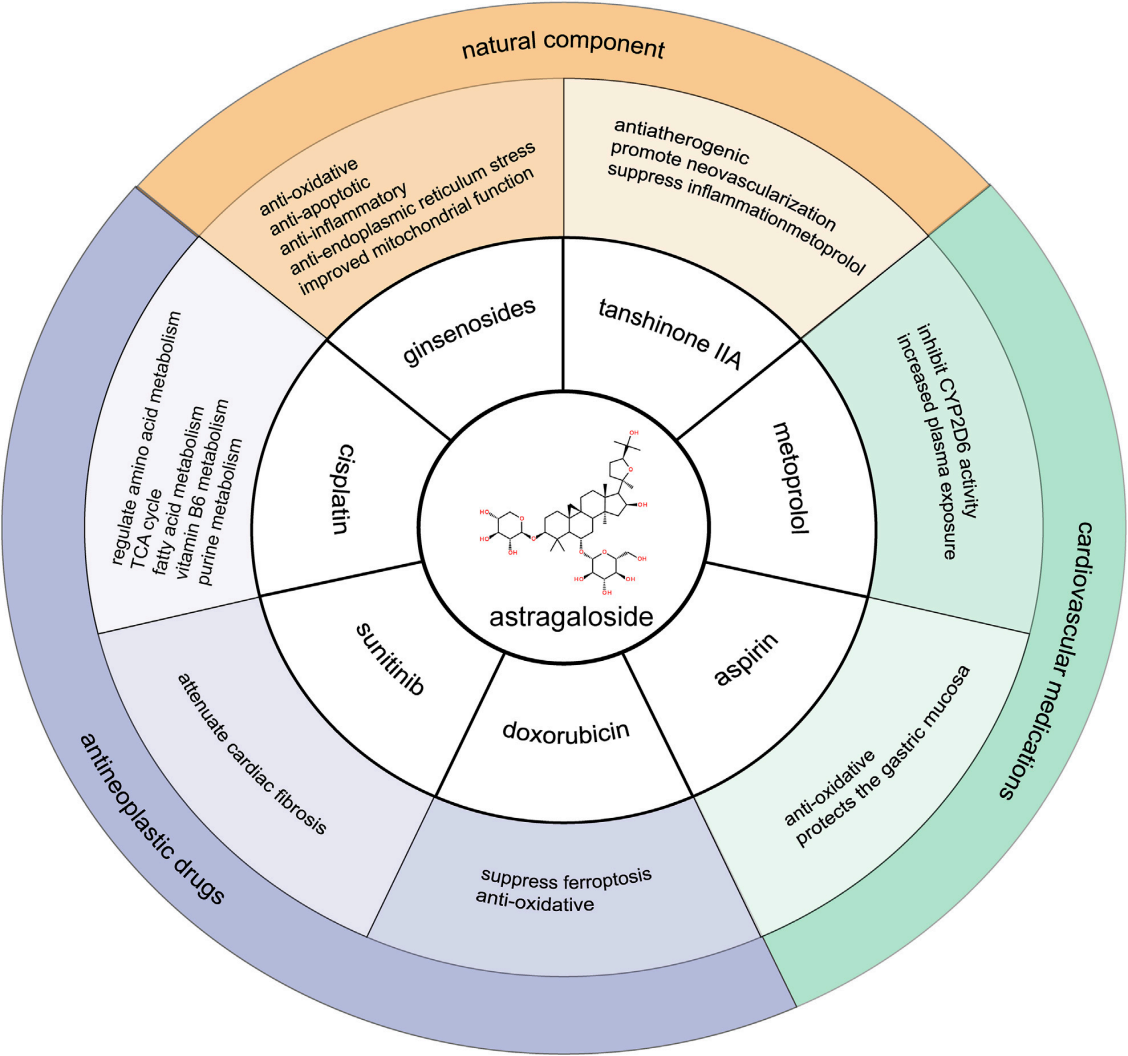
Synergistic interactions of astragaloside IV with other medications.

The therapeutic potential of AS-IV extends beyond its individual effects, particularly in its synergistic interactions with other natural compounds and commonly used pharmacological agents. This section examines the combined efficacy of AS-IV with other TCM components, as well as its ability to reduce side effects when used in conjunction with cardiovascular drugs such as metoprolol and aspirin. Additionally, the potential of AS-IV to mitigate toxicity when co-administered with anticancer drugs is explored, highlighting its broader therapeutic applicability and role in optimizing polypharmacy strategies for improved patient outcomes (Figure 2).

### 4.1 The active components of natural medicines

#### 4.1.1 The ginsenoside compounds

Ginsenosides, recognized as the primary anti-arrhythmic agents in ginseng extracts, function by blocking various ion channels (Sun et al., 2016). Research indicates that combining AS-IV with ginsenoside Rg1, ginsenoside Rb1, or notoginsenoside R1 provides superior protection against cerebral ischemia/reperfusion (I/R) injury through multiple mechanisms compared to the effects of individual compounds. Specifically, when schisandrin IV is co-administered with ginsenoside Rg1, Rb1, or notoginsenoside R1, it exhibits significant activation of the Nrf2/HO-1 signaling pathway after cerebral I/R, which is not observed with its individual application. This combination downregulates Nrf2 in the cytoplasm while upregulating its nuclear expression, enhancing nuclear translocation and increasing HO-1 mRNA and protein expression. As a result, the combined treatment demonstrates a markedly improved antagonistic effect against I/R-induced oxidative stress (Huang et al., 2014).

Furthermore, co-administration of AS-IV with these ginsenosides reduces neuronal apoptosis in injured brain tissue by downregulating caspase-3 protein expression in the CA1 hippocampal region of model rats. In addition, this combination not only inhibits inhibitor of nuclear factor kappa B (NF-κB) phosphorylation, thereby reducing nuclear translocation of NF-κB and suppressing downstream inflammatory cytokine expression (TNF-α and ICAM-1 mRNA) within the NF-κB signaling pathway but also mitigates phosphorylation of Janus Kinase 1 (JAK1) and signal transducer and activator of transcription 1 (STAT1), suppressing activation of the JAK1/STAT1 pathway to enhance inflammatory response inhibition post-cerebral ischemia. Finally, the joint application of AS-IV with these compounds significantly reduces caspase-12 expression in brain tissue after I/R, inhibits phosphorylation of JNK1/2 proteins, and increases GRP78 protein expression. Consequently, this treatment alleviates the accumulation of unfolded or misfolded proteins associated with endoplasmic reticulum stress (ERS), thereby mitigating ERS-induced injury following I/R (Huang et al., 2015).

Although studies exploring the anti-arrhythmic potential of these combined monomeric components are limited, research on the combined use of astragalus and ginseng has shown promise. For instance, Wu et al. reported that the Tongyang Huoxue Formula enhances ion channel function in the sinoatrial node (Wu et al., 2024), while Chang et al. demonstrated that the Zishen Tongyang Huoxue Formula ameliorates I/R injury in sinoatrial node cells by regulating mitochondrial quality control through the VDAC1–β-tubulin signaling axis (Chang et al., 2024).

#### 4.1.2 Tanshinone IIA

The traditional Chinese medicinal herb *Salvia miltiorrhiza* (Danshen) is extensively used for the treatment of cardiovascular diseases, with Tanshinone IIA as its primary active constituent. According to the principles of TCM, combining *Astragalus membranaceus* with *S. miltiorrhiza* is believed to enhance the blood circulation-promoting and blood stasis-eliminating effects of Danshen. Recent research has shown that moderate angiogenesis following MI can effectively reduce cardiomyocyte mortality, increase collateral vessel density, maintain ejection fraction, and prevent adverse cardiac remodeling (Chen et al., 2017; Cheng et al., 2019; Zhu et al., 2021). Endothelial cell proliferation and migration are critical steps in the angiogenesis process.

Li et al. demonstrated that the combined administration of AS-IV and tanshinone IIA significantly upregulated the expression of gap junction proteins Cx37, Cx40, and Cx43 in mesenchymal stem cell-derived endothelial-like cells. This enhancement in gap junction intercellular communication facilitated the proliferation and angiogenic potential of these endothelial-like cells. Furthermore, experimental findings indicated that the expression levels of Cx37, Cx40, and Cx43 in the combination treatment group were significantly higher than in groups treated with AS-IV or tanshinone IIA alone (Li et al., 2018).

Aortic atherosclerotic plaques constitute an independent risk factor for stroke in patients with AF, making plaque stabilization crucial for stroke prevention and management in these individuals. Wang et al. reported that, compared to the effects of either compound alone, the combined treatment with AS-IV and tanshinone IIA more effectively reduced lipid areas in the right common carotid artery, increased collagen content, thickened the fibrous cap, and enhanced plaque stability. The primary mechanism involves activation of the PI3K/AKT signaling pathway, which alleviates oxidative stress in vascular endothelial cells, while concurrently inhibiting the TLR4/NF-κB signaling pathway. This inhibition decreases the nuclear translocation of NF-κB, leading to the downregulation of inflammatory mediators such as TLR4, IL-6, matrix metalloproteinase 9, TNF-α, and CRP (Wang et al., 2020).

### 4.2 Optimization for minimizing side effects of cardiovascular drugs

#### 4.2.1 Metoprolol

AS-IV has demonstrated potential interactions with antiarrhythmic agents, including metoprolol. Research findings suggest that AS-IV inhibits the activity of cytochrome P450 2D6 (CYP2D6), thereby slowing the metabolism of metoprolol and increasing its plasma concentration. Consequently, a reduction in the dosage of metoprolol may be warranted (Shi et al., 2022). Additionally, AS-IV can competitively inhibit CYP1A2 activity, subsequently decreasing the clearance rate of theophylline (Zhang et al., 2013). These findings indicate that AS-IV may reduce the metabolic processing of antiarrhythmic drugs, thereby lowering the required clinical dosage and enhancing therapeutic efficacy.

#### 4.2.2 Aspirin

Aspirin is a cornerstone of anti-platelet therapy for cardiovascular thrombotic disorders (Kim et al., 2023). Chronic low-dose aspirin therapy irreversibly inhibits the cyclooxygenase-1 **(**COX-1) activity in platelets, leading to reduced thromboxane A2 production and thereby exerting anti-platelet aggregation effects. This mechanism plays a critical role in the prevention and management of cardiovascular thrombotic diseases (Schrör, 1997). However, long-term aspirin use, particularly in individuals with pre-existing gastric conditions, is associated with damage to the gastric mucosa, ranging from mild mucosal erosion and ulceration to more severe complications, such as bleeding or perforation. Even with the use of enteric-coated formulations at low doses, the risk of gastric inflammation and bleeding remains significant (Pugliese and Taddei, 2023). As a result, many patients on long-term aspirin therapy are required to co-administer gastric-protective agents or switch to alternative anti-platelet drugs, which imposes substantial limitations on its clinical use.

Research conducted by Fan et al. (2016) demonstrated that the combination of aspirin and AS-IV in rat models of gastritis was effective in mitigating the gastric mucosal ulcers induced by aspirin. The proposed mechanism underlying this effect may involve the ability of AS-IV to counteract the inhibitory effect of aspirin on COX-1 expression in gastric mucosal tissues (Fan et al., 2016). Additionally, AS-IV was found to promote the synthesis of prostaglandin E2, enhance the activities of superoxide dismutase-1 and nitric oxide, and protect the gastric mucosa from oxidative stress-induced damage (Fan et al., 2016). These findings suggest that AS-IV could serve as a promising therapeutic option for alleviating the gastric side effects of long-term aspirin use. However, further investigation is needed to determine whether AS-IV mitigates with the anti-platelet effects of aspirin.

### 4.3 Alleviation of the toxic side effects of drugs

#### 4.3.1 Antineoplastic drugs

Anti-cancer agents, including alkylating agents, human epidermal growth factor receptor two receptor blockers, anthracyclines, microtubule modulators, tyrosine kinase inhibitors, histone deacetylase inhibitors, and antimetabolites, are collectively associated with significant cardiotoxicity. These agents can lead to the development of arrhythmias, with AF being the most common manifestation. The adverse effects of anti-cancer drugs extend to the functional capacity of various cardiac cell types, including cardiac myocytes, fibroblasts, and endothelial cells. These drugs disrupt the autocrine and paracrine signaling pathways between these cells, ultimately resulting in alterations to cardiac cell homeostasis (Koukorava et al., 2024). AS-IV has been shown to play a detoxifying role in anti-tumor therapy through multiple mechanisms.

#### 4.3.2 Doxorubicin

Doxorubicin (DOX), a widely employed chemotherapeutic agent, is nonetheless limited in its clinical application due to its significant cardiotoxicity. In a rat model of DOX-induced cardiotoxicity, it has been unequivocally demonstrated that DOX induces the production of ROS and transforming growth factor-beta (TGF-β), thereby promoting the transformation of cardiac fibroblasts into myofibroblasts (Cappetta et al., 2016). Research indicates that AS-IV can mitigate DOX-induced cardiac injury through several mechanisms. Specifically, AS-IV has been shown to reverse the reduction in SIRT1 levels induced by DOX in mice, while inhibiting the activation of the NLRP3 inflammasome, thereby reducing cellular apoptosis and improving cardiac function (Tian et al., 2024). Furthermore, AS-IV may activate the PI3K/Akt signaling pathway to suppress oxidative stress, contributing to the amelioration of DOX-induced cardiac dysfunction (Jia et al., 2014; Luo et al., 2021b). The study also revealed that AS-IV could alleviate iron-dependent cell death in cardiomyocytes by activating the Nrf2 signaling pathway and enhancing the expression of GPx4, thereby attenuating oxidative stress induced by DOX (Luo et al., 2021a). In addition, AS-IV has been found to restore the expression of p62, a sortilin protein, by reducing the levels of cystatin-C (Cyt-C) and poly (ADP-ribose) polymerase (PARP), thus correcting the autophagy imbalance in cardiomyocytes caused by DOX (Luo et al., 2021b). These findings suggest that AS-IV holds considerable potential for mitigating DOX-induced cardiotoxicity.

#### 4.3.3 Sunitinib

Sunitinib (SU) is a widely used anti-tumor agent; however, its clinical application is often limited by its associated cardiac toxicity, which frequently leads to drug discontinuation. Studies have demonstrated that AS-IV can effectively inhibit the myocardial inflammatory infiltration and fibrotic lesions induced by SU, as well as mitigate oxidative stress and apoptosis in cardiomyocytes. AS-IV alleviates myocardial damage caused by SU through the downregulation of COUP-TFII expression and improves cardiac function by attenuating cardiac fibrosis (Qin et al., 2024). These findings suggest that AS-IV holds potential as an adjunctive agent in anti-tumor therapies, offering protection against the cardiac side effects, such as arrhythmia, that are commonly associated with SU treatment.

#### 4.3.4 Cisplatin

Cisplatin is a commonly used chemotherapy agent for the treatment of various cancer types; however, its clinical utility is often constrained by its toxicity, particularly in non-cancerous tissues. Research has shown that AS-IV significantly inhibits the growth of colorectal cancer cells while exhibiting minimal toxic effects on non-malignant colon cells (Xie et al., 2016). Moreover, AS-IV enhances the chemosensitivity of colorectal cancer cells to cisplatin, with part of its mechanism involving the suppression of NOTCH3 expression (Xie et al., 2016). Urinary metabolomics analyses have further indicated that Huang-qi injections can modulate amino acid metabolism, the tricarboxylic acid (TCA) cycle, fatty acid metabolism, vitamin B6 metabolism, and purine metabolism in cisplatin-induced nephrotoxic rats (Liu et al., 2019). This sensitizing effect suggests that AS-IV may help mitigate cisplatin-induced toxicity by reducing the required dosage of the chemotherapeutic agent, thereby alleviating cardiotoxicity during cisplatin therapy.

Through comprehensive investigations into the cardiotoxicity of various anti-tumor drugs, AS-IV has demonstrated a broad capacity to attenuate toxicity. Its protective effects involve multiple mechanisms, including antioxidation, suppression of inflammation, modulation of autophagy, and enhancement of calcium homeostasis, which collectively safeguard cardiomyocytes. Additionally, the detoxifying properties of AS-IV may indirectly reduce the risk of arrhythmia induced by these drugs, thus providing a promising strategy to improve the safety and efficacy of anti-tumor treatments.

## 5 AS-IV modulates the risk factors associated with arrhythmia

**FIGURE 3:**
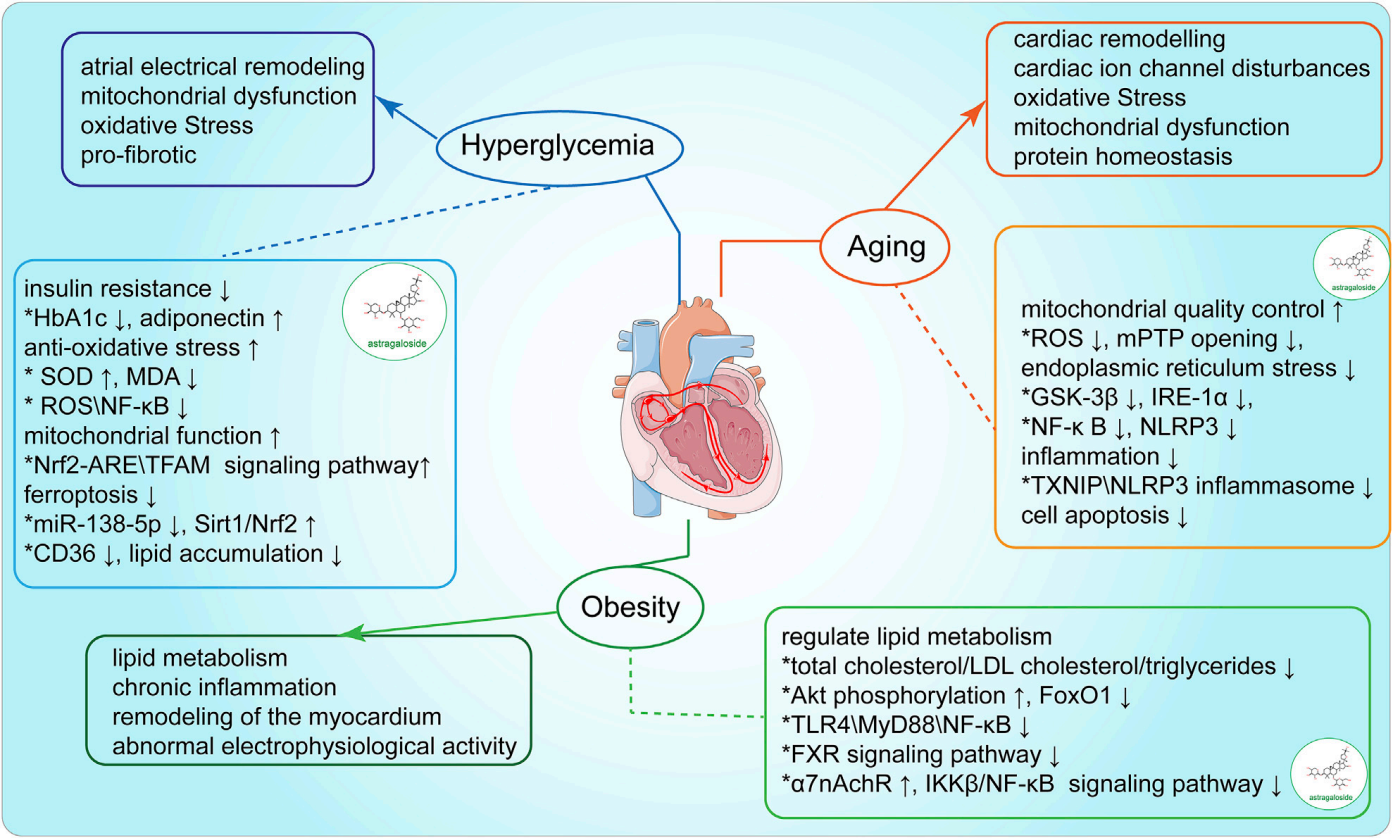
Modulation of arrhythmia-associated risk factors by AS-IV

Arrhythmia is often triggered or exacerbated by various risk factors, including aging, blood glucose dysregulation, and obesity. These risk factors have been strongly associated with arrhythmia in clinical observations. AS-IV has been shown to exert a range of pharmacological effects, including antioxidant, anti-inflammatory, and cardioprotective actions, which can mitigate the damage caused by these risk factors. This section explores the mechanisms by which AS-IV influences the key risk factors underlying arrhythmia, with the potential to exert therapeutic effects by controlling the impact of these risk factors (Figure 3).

### 5.1 Anti-aging

As age progresses, the risk of cardiovascular diseases, including atrial and ventricular arrhythmias, rises significantly. Clinical studies have established a robust correlation between biological aging and AF incidence. In a study of 5,600 participants with an average age of 65.5 years, epigenetic clocks (e.g., DNAm GrimAge and DNAm PhenoAge) were utilized to assess biological age. Findings indicated that each 5-year increase in epigenetic age was associated with a 19% increase in AF risk, while acceleration of DNAm PhenoAge was linked to a 15% rise in AF risk (Roberts et al., 2021). The aging process leads to reduced T-tubule density in cardiomyocytes, potentially facilitating post-depolarization phenomena, reentry events, and alternans, thereby establishing an electrophysiological basis for arrhythmia (Setterberg et al., 2021). Although the mechanisms underlying arrhythmia in older adults remain incompletely understood, cardiac structural remodeling is a recognized contributing factor (Kane et al., 2021). In aged rabbit models, senescent cardiac fibroblasts couple with cardiomyocytes, resulting in prolonged action potential duration (APD), conduction block, and an increased arrhythmia risk (Baggett et al., 2023). Furthermore, telomere shortening during aging is closely associated with atrial remodeling linked to AF. In a cohort of 155 patients with paroxysmal AF, with a mean age of 56.8 years, individuals with significant reductions in leukocyte telomere length exhibited a higher recurrence rate of AF (HR 2.68, 95% CI 1.50–4.79, P = 0.001), along with more pronounced left atrial enlargement, indicating severe atrial remodeling (Pan et al., 2019).

Research by Tahhan et al. demonstrated that an increase in redox potential (elevation of EhGSH) is significantly associated with both the prevalence and incidence of AF. Specifically, a 10% rise in EhGSH corresponded to a 30% increased likelihood of developing AF and a 40% rise in episode occurrence risk, highlighting the critical role of oxidative stress in AF pathogenesis (Samman Tahhan et al., 2017). Oxidative stress is recognized as a pivotal aging mechanism. With advancing age, mitochondrial function deteriorates, characterized by increased ROS production, accumulation of mitochondrial DNA (mtDNA) mutations, altered mitochondrial dynamics, a decline in the mitochondrial unfolded protein response (UPRmt), and reduced respiratory chain activity (Bao et al., 2023). Recent studies indicate that AS-IV effectively mitigates high-glucose-induced cellular senescence by promoting mitochondrial autophagy, maintaining mitochondrial dynamic homeostasis, and enhancing energy metabolism (Jun-li et al., 2023; Sun et al., 2023) Additionally, AS-IV reduces ROS overproduction and mitochondrial dysfunction induced by angiotensin II (Ang II), thereby inhibiting vascular smooth muscle cell senescence (Lu Y. et al., 2015).

Inflammatory responses also play a key role in cellular senescence. Interleukin-6 (IL-6) regulation is considered to be independent of the aging-related gene P53 and instead is modulated directly by the DNA damage response (DDR), influencing the IL-6 signaling complex expression on cell surfaces (Rodier et al., 2009). Similarly, interleukin-1 (IL-1) protein is upregulated during cellular senescence, enhancing NF-κB-mediated inflammatory cascade activation (Lau et al., 2019). In a study involving 72 patients with chronic heart failure (CHF), Astragalus injection significantly reduced inflammatory cytokines, including tumor necrosis factor-alpha (TNF-α) and IL-6, in elderly CHF patients (Zhang et al., 2005). Experimental evidence indicates that AS-IV downregulates advanced glycation end product (AGE) expression in hippocampal tissue in aging model rats, thereby inhibiting AGE-RAGE protein interactions on neurons and inflammatory cells, suppressing NF-κB pathway activation, and reducing inflammation-related factors such as IL-6, IL-1β, and TNF-α. This effect alleviates hippocampal neuronal inflammation and improves memory deficits in D-gal-induced aging model rats (Li W. et al., 2022).

Protein homeostasis is intimately linked with aging and longevity. Biomarkers of aging include the status of chaperone proteins, autophagy-lysosome and ubiquitin-proteasome system activities, and UPRER and UPRmt functionality (Henning and Brundel, 2017). Recent studies reveal that ERS, strongly correlated with protein homeostasis, is closely linked to arrhythmia. The relationship between the endoplasmic reticulum and mitochondria is essential in combating cellular apoptosis and stress. Studies indicate that 50 μM AS-IV significantly inhibits GSK-3β kinase activity in 2-DG-induced PC12 cells, prevents mitochondrial permeability transition pore (mPTP) opening, downregulates IRE1 and p-PERK, and thus mitigates mitochondrial damage and apoptosis under ERS (Fu et al., 2020). In diabetic nephropathy models, the AS-IV group showed significantly reduced expressions of GRP78 and IRE-1α, key ERS regulators, resulting in suppression of NF-κB/NLRP3 pathway activation, downregulation of NF-κB p65 and IL-1β, and subsequent inflammation and podocyte apoptosis mitigation in diabetic nephropathy rats (Sun et al., 2024). Similarly, Zhao et al. showed that AS-IV inhibits IRE-1α/TXNIP/NLRP3 signaling, reduces ROS production, and downregulates caspase-1 and IL-1β, alleviating oxidative stress and inflammation associated with ERS, and reducing endothelial cell apoptosis. AS-IV also activates AMP-activated protein kinase (AMPK), promotes TXNIP degradation, and inhibits TXNIP activity, protecting endothelial cells from TXNIP/NLRP3 inflammasome-induced damage (Zhao Y. et al., 2015). Although current research primarily focuses on non-cardiac diseases, these findings highlight AS-IV’s potential in modulating ERS in cardiac arrhythmias.

Cellular senescence is dynamic, often compromising normal tissue functionality by continuously secreting pro-inflammatory cytokines, chemokines, growth factors, and matrix metalloproteinases—collectively termed senescence-associated secretory phenotype (SASP) factors. SASP factors mediate chronic inflammation, activate cardiac fibroblasts, and exacerbate cellular senescence and tissue damage (Huang et al., 2022; Mehdizadeh et al., 2022). Aging cardiomyocytes significantly increase SASP factor expression, including CCN1, ILs (1α, 1β, and 6), TNF-α, MCP-1, TGF-β, and GDF15, which are closely associated with age-related cardiovascular dysfunction (Mehdizadeh et al., 2022). For instance, senescent M1 macrophages within atherosclerotic plaques release SASPs, increasing oxidative stress levels within plaques, leading to instability, rupture, and a heightened risk of AF (Vellasamy et al., 2022). Recent studies demonstrate that AS-IV mitigates isoproterenol (ISO)-induced myocardial injury and fibrosis by suppressing SASP release and modulating the p53 signaling pathway (Shi et al., 2024). AS-IV also supports the function of key ion channels (e.g., HCN4) in the heart by enhancing anti-aging protein Klotho expression, reducing arrhythmia likelihood (Qiu et al., 2016).

### 5.2 Regulation of blood glucose

Hyperglycemia is recognized as an independent risk factor for the onset of AF. A study of 8,943 patients with impaired glucose tolerance demonstrated that for every 1 mmol/L increase in fasting blood glucose, the risk of AF increased by 33%, indicating that elevated blood glucose levels may contribute to AF even before diabetes is diagnosed (Latini et al., 2013). Compared to the general population, patients with diabetes and those with stage 5 CKD show a threefold higher risk of developing AF (Seyed Ahmadi et al., 2020). Additionally, the presence of diabetes exacerbates the bleeding risk in patients with AF (Chan et al., 2020). Diabetes-induced oxidative stress and inflammation are crucial factors in the pathogenesis of AF. Hyperglycemia impairs mitochondrial function, leading to excessive production of ROS (Yuan et al., 2020), which directly damages cardiomyocytes, causing atrial fibrosis and abnormal electrical activities (Liang et al., 2018). ROS further contributes to myocardial structural and electrical remodeling by promoting the release of inflammatory factors such as TNF-α and IL-6. Chronic inflammation, in turn, enhances the expression of pro-fibrotic factors via the activation of pathways like NF-κB, damaging atrial intercellular junction proteins (e.g., Cx40 and Cx43) and affecting electrical signal transmission in the myocardium (Karam et al., 2017). The combined effects of oxidative stress and inflammation facilitate atrial fibrosis and electrical remodeling in diabetic patients, thereby increasing the risk of AF.

AS-IV shows significant potential in glycemic control. In a randomized controlled trial targeting type 2 diabetes mellitus patients with chronic kidney disease, Astragalus administration significantly reduced fasting blood glucose and HbA1c levels and improved insulin resistance (Chan et al., 2024). Liang et al. reported that Astragalus combined with insulin markedly increased SOD levels, decreased MDA content, reduced oxygen free radical generation, and enhanced antioxidant activity in pancreatic islet cells, thus effectively lowering blood glucose levels in patients with gestational diabetes (Liang et al., 2009). Xu et al. found that AS-IV improved hyperglycemia, insulin resistance, and glucose tolerance in diet-induced and genetically obese mice by elevating circulating levels of adiponectin (Xu et al., 2009).

AS-IV has demonstrated efficacy in alleviating diabetes and its complications through antioxidant, anti-inflammatory, anti-fibrotic, and anti-ferroptotic mechanisms. A meta-analysis on Astragalus and its extracts in stage III-IV diabetic nephropathy revealed that Astragalus inhibited early macrophage-induced nitric oxide synthase activity triggered by lipopolysaccharide, reduced NO and TNF-α production, and decreased apoptosis in renal tubular epithelial cells (Lai et al., 2013; Wang et al., 2016; Liao et al., 2017). Furthermore, Su et al. showed that AS-IV activates the PI3K/AKT/Nrf2/ARE signaling pathway, enhancing Nrf2 expression and nuclear translocation, binding to the ARE upstream, and increasing downstream antioxidant protein expression (e.g., Ho-1, Gpx-1, Gclc, and Gclm), which mitigates oxidative stress in glomerular mesangial cells (GMCs) induced by high glucose. This study further revealed that AS-IV can limit NF-κB nuclear translocation in high-glucose-stimulated GMCs by upregulating Nrf2 and reducing ROS generation, subsequently downregulating inflammatory markers like MCP-1, TNFα, IL-1β, IL-6, and fibrosis markers Fn and Col IV downstream of NF-κB, thereby ameliorating GMC inflammation and fibrotic damage under high glucose (Su et al., 2023). Deng et al. demonstrated that AS-IV reduces blood glucose levels and increases serum insulin in juvenile mice with diabetic ketoacidosis (DKA). AS-IV also enhances catalase and glutathione peroxidase secretion in pancreatic tissue, degrading H2O2, preventing ROS interactions, and thereby increasing SOD activity and reducing MDA levels, which mitigates oxidative stress damage in DKA pancreatic tissue through the activation of the JNK/Nrf2 signaling pathway. Notably, when JNK and Nrf2 expression is silenced, AS-IV’s protective effects on pancreatic oxidative stress in DKA are diminished, though AS-IV still improves glucose and insulin levels in these juvenile mice (Deng, 2020). Additionally, AS-IV mitigates myocardial dysfunction in diabetic cardiomyopathy rats by downregulating CD36-mediated ferroptosis (Li et al., 2023). Tang et al. demonstrated that AS-IV alleviates ferroptosis in retinal pigment epithelial cells under high glucose by modulating the miR-138–5p/Sirt1/Nrf2 signaling pathway (Tang et al., 2022).

These findings suggest that AS-IV may improve metabolic status, reduce oxidative stress and inflammation, and potentially aid in the prevention or delay of diabetic complications such as AF.

### 5.3 The control of obesity

Obesity is not only a well-established cardiovascular risk factor but also significantly increases the risk of sudden cardiac death and AF. Research has shown that the recurrence rate of AF is 20% in patients who undergo bariatric surgery, compared to as high as 55% in morbidly obese individuals who do not undergo such interventions (Donnellan et al., 2019). In the renowned Framingham Heart Study, a detailed analysis of clinical data from 5,204 participants revealed that the risk of developing new-onset AF among obese individuals was twice as high as that in individuals of normal weight. Furthermore, for each unit increase in body mass index, the risk of AF rose by 7% in men and 9% in women. Left atrial enlargement resulting from obesity was identified as the primary mediator of AF, underscoring a significant correlation between obesity and atrial structural remodeling (Parish, 2005). Obesity contributes to the creation of substrates conducive to arrhythmia (Pathak et al., 2015), facilitating the onset and progression of AF. These substrates include lipid metabolism disorders, elevated blood pressure, chronic inflammation, cardiac structural remodeling, and electrophysiological abnormalities (Lavie et al., 2017; Middeldorp et al., 2023). Therefore, controlling obesity is crucial for mitigating the risk of arrhythmias.

Previous studies have demonstrated that AS-IV is effective in managing obesity and reducing body weight. A study involving 84 patients with gestational diabetes showed that, compared to insulin therapy alone, the combination of insulin and astragalus significantly reduced levels of total cholesterol, triglycerides, and low-density lipoproteins (Liang et al., 2009). AS-IV notably reduced body weight in aged mice, decreased white adipose tissue, and lowered the liver-to-body weight ratio. It also significantly decreased triglyceride (TG) levels in both serum and liver, enhanced fatty acid mobilization in white adipose tissue, promoted mitochondrial fatty acid oxidation, and facilitated mitochondrial biogenesis in the liver (Luo Z. et al., 2021).

AS-IV modulates the Akt/PDE3B pathway to inhibit cAMP accumulation, thereby suppressing lipid catabolism, which is beneficial for limiting liver lipid deposition and reducing excessive hepatic glucose production (Du et al., 2018). Additionally, AS-IV activates AMPK to alleviate ERS and lipid accumulation induced by free fatty acids in hepatocytes, showing potential therapeutic efficacy in hepatic steatosis (Zhou et al., 2017). Liang et al. found that AS-IV significantly reduced levels of AST, ALT, and TG in the serum of non-alcoholic fatty liver disease mice (Liang et al., 2021). Further studies suggested that the mechanism might involve the suppression of the TLR4/MyD88/NF-κB signaling pathway by AS-IV, which downregulates the expression of downstream inflammatory cytokines such as TNF-α, IL-6, and IL-8, thereby alleviating the inflammatory response induced by liver steatosis (Liu et al., 2020).

AS-IV also regulates blood glucose and lipid metabolism through modulation of the gut microbiota. Studies have shown that AS-IV downregulates the expression of intestinal fibroblast growth factor 15, activates the liver farnesoid X receptor (FXR) signaling pathway, upregulates glucagon-like peptide-1, and reduces ceramide production, thereby modulating lipid synthesis processes that regulate cholesterol transport and optimize lipid metabolism in the liver (Zhai et al., 2022). Moreover, AS-IV has been shown to mitigate obesity-related hypertension by repressing inflammatory responses and enhancing leptin resistance, likely through upregulation of α7nAchR levels and inhibition of IKKβ/NF-κB signaling in the hypothalamus and adipose tissue (Jiang et al., 2018).

Given the strong link between obesity and arrhythmia, the aforementioned studies suggest that astragaloside may reduce the likelihood of arrhythmia by regulating fat metabolism. While a considerable body of basic research has revealed the potential beneficial effects of astragalus on obesity-related arrhythmias, clinical evidence directly demonstrating its efficacy in obesity management remains limited. Therefore, further clinical trials are essential to validate the precise therapeutic potential of astragaloside in obesity management, which will provide stronger support for its use in patients with arrhythmia.

## 6 Conclusion

AS-IV, a bioactive compound derived from *Astragalus membranaceus*, demonstrates significant cardioprotective effects, particularly in the management of arrhythmias. Through multiple pathways, AS-IV effectively mitigates myocardial ischemia-reperfusion injury, modulates calcium homeostasis, suppresses inflammatory responses, and counteracts oxidative stress. Furthermore, the co-administration of AS-IV with other TCM components or anti-arrhythmic agents has shown to enhance bioavailability and therapeutic efficacy, thereby expanding its potential clinical applications.

However, most existing studies are primarily based on animal models, with limited clinical trial data available. Several factors contribute to the absence of clinical studies on AS-IV in arrhythmia treatment. One major obstacle is the difficulty in translating animal model results into human clinical settings due to species-specific variations in drug metabolism, pharmacokinetics, and the complexity of arrhythmias in human patients. Additionally, the bioavailability of AS-IV in oral formulations remains a challenge, as the compound is poorly absorbed, which hinders its therapeutic potential in humans.

To overcome these challenges, several strategies can be considered. Firstly, enhancing the pharmacokinetic properties of AS-IV through advanced drug delivery systems, such as nanocarriers or liposomal formulations, may improve its bioavailability and ensure more consistent therapeutic levels. Secondly, the identification of specific molecular targets and pathways through which AS-IV exerts its effects could facilitate the design of targeted clinical trials, allowing for a more precise understanding of its mechanisms of action. Finally, conducting multi-center, randomized clinical trials with larger sample sizes and longer follow-up periods will be essential to confirm the clinical efficacy and safety of AS-IV in arrhythmia treatment, especially in patients with varying underlying pathologies.

Future research should focus on optimizing the bioavailability of AS-IV in oral formulations, elucidating its mechanisms of action within multi-drug combination therapies, and evaluating its clinical efficacy across varied pathological conditions. These endeavors aim to establish a more comprehensive treatment protocol for arrhythmia patients.
